# Light–matter interactions in two-dimensional layered WSe_2_ for gauging evolution of phonon dynamics

**DOI:** 10.3762/bjnano.11.63

**Published:** 2020-05-12

**Authors:** Avra S Bandyopadhyay, Chandan Biswas, Anupama B Kaul

**Affiliations:** 1 Department of Electrical Engineering, University of North Texas, Denton, TX 76203, United States; 2 Department of Materials Science and Engineering; PACCAR Technology Institute; University of North Texas, Denton, TX 76203, United States; 3 Department of Electrical and Computer Engineering, University of Texas, El Paso, TX 79968, United States

**Keywords:** phonon concentration, phonon lifetime, Raman spectroscopy, thermal coefficients, Tungsten diselenide, two-dimensional material

## Abstract

Phonon dynamics is explored in mechanically exfoliated two-dimensional WSe_2_ using temperature-dependent and laser-power-dependent Raman and photoluminescence (PL) spectroscopy. From this analysis, phonon lifetime in the Raman active modes and phonon concentration, as correlated to the energy parameter *E*_0_, were calculated as a function of the laser power, *P*, and substrate temperature, *T*. For monolayer WSe_2_, from the power dependence it was determined that the phonon lifetime for the in-plane vibrational mode was twice that of the out-of-plane vibrational mode for *P* in the range from 0.308 mW up to 3.35 mW. On the other hand, the corresponding relationship for the temperature analysis showed that the phonon lifetime for the in-plane vibrational mode lies within 1.42× to 1.90× that of the out-of-plane vibrational mode over *T* = 79 K up to 523 K. To provide energy from external stimuli, as *T* and *P* were increased, peak broadening in the PL spectra of the *A*-exciton was observed. From this, a phonon concentration was tabulated using the Urbach formulism, which increased with increasing *T* and *P*; consequently, the phonon lifetime was found to decrease. Although phonon lifetime decreased with increasing temperature for all thicknesses, the decay rate in the phonon lifetime in the monolayer (1L) material was found to be 2× lower compared to the bulk. We invoke a harmonic oscillator model to explain the damping mechanism in WSe_2_. From this it was determined that the damping coefficient increases with the number of layers. The work reported here sheds fundamental insights into the evolution of phonon dynamics in WSe_2_ and should help pave the way for designing high-performance electronic, optoelectronic and thermoelectric devices in the future.

## Introduction

Since the discovery of graphene, atomically thin two-dimensional layered materials have drawn intense attention due to their unique physical properties [1–2]. Two-dimensional (2D) layered materials beyond graphene, such as transitional metal dichalcogenides (TMDCs) [3], black phosphorus (BP) [4], and other families of layered materials [5], can be mechanically exfoliated or, in some cases, grown from bottom-up processes akin to graphene. While graphene is comprised of a single element on the periodic table, i.e., carbon, TMDCs are binary compounds which makes their lattice dynamics more complex compared to multilayer (ML) graphene [6]. The symmetry, force constants, and frequency variation with geometrical confinement in some TMDCs has been studied recently [7]. Monolayer (1L) TMDCs consist of a plane of a transition metal, M, sandwiched by chalcogenides, X, on either side to yield the stoichiometry MX_2_ [8]. The interlayer bonding in most ML TMDCs is through the weak van der Waals interaction while the intra-layer bonding is via the strong covalent interaction. This makes them inherently flexible and good candidates for flexible electronics [9], optoelectronics [10], and other related applications [11–12]. Amongst the TMDCs, WSe_2_ offers unique advantages for device applications, which includes its high mobility of ≈500 cm^2^/V·s at room temperature, and a strong spin–orbit coupling [3,13–14]. Thus, it is not surprising that a rich variety of electronic and optoelectronic devices have already been demonstrated using 1L WSe_2_ which harnesses its exceptional properties [13,15–16].

It is well-understood that the underlying factors governing the optical, electronic and thermal properties of solid-state materials are strongly influenced by phonons and their spatio-temporal response toward external stimuli. Raman and photoluminescence (PL) spectroscopy has been a remarkable tool to gauge phonon dynamics for a broad range of materials in the past, including nanocarbons [17]. Phonon dynamics in 2D TMDCs, just as in other materials, includes discerning factors such as phonon lifetime τ and the change in phonon concentration as determined from the characteristic energy parameter *E*_0_, calculated from the slope of the low-energy edge of the excitonic mode of the PL spectra. The theory of spectral line shape in the Raman spectrum predicts a Lorentzian distribution of a collected signal in a dispersive medium, where the full-width-half-maximum (FWHM) scales as 1/τ, and not surprisingly, τ is influenced by damping mechanisms. The FWHM is expected to be infinitesimally small for activated phonons in a dissipationless medium, and the crystal elastic waves of the harmonic oscillator model for the allowable phonon modes would thus yield an exceptionally large τ. However, natural systems inherently exhibit damping, and thus the FWHM of the Raman peaks have a finite width, indicating the presence of decay channels that reduce τ. In general, the phonon linewidths contain contributions arising from several scattering mechanisms such as the electron–electron interaction, i.e., Coulombic scattering, or the electron–phonon interaction, i.e., scattering of electrons from defects. Chakrabarty et al. reported that the linewidth of the *A*_1g_ peak in single-layer MoS_2_ that was subsequently used in transistors, broadened due to *n*-type doping where the phonon linewidth renormalized under the presence of an electric filed [18]. Similarly, the Raman linewidths in graphene are found to increase with defects resulting from electron–impurity and electron–phonon scattering [19]. Moreover, the Raman linewidth broadening is also attributed to the confinement of the optical phonons. Specifically, in the case of low-dimensionality nanocrystallites, the wave function of the optical phonons no longer remains a continuous plane wave and thus the localization of the wave function leads to a relaxation in the conservation of the wave vector selection rules. The phonons with a nonzero wave vector also take part in the Raman scattering process along with the phonons with zero wave vector, which results in broadening of the phonon linewidths. Another important parameter in this analysis is the actual position of the allowable Raman modes, which are typically the higher energy optical phonons, and how these modes interact with external stimuli. For example, external radiation could be in the form of heat or optical energy, which also directly influences properties such as the electronic and optoelectronic transport and the thermal conductivity of the material.

In this work, we have conducted an in-depth analysis of the phonon dynamics in WSe_2_, where τ and the change in phonon concentration deciphered from an energy parameter *E*_0_ were quantified as a function of external stimuli, specifically temperature, *T*, of the WSe_2_ and the Raman laser power, *P*, in 1L, ML and bulk WSe_2_ samples using Raman and PL spectroscopy. The temperature dependence of the Raman shifts in 2D TMDCs such as MoS_2_ [20–24], and WS_2_ [25–26] have been extensively studied over a wide temperature range from which properties such as thermal conductivity was deciphered [23,27]. On the contrary, the temperature-dependent Raman analysis of WSe_2_ is rather limited with only one prior report discussing the thermal coefficients of various Raman modes in WSe_2_ where a comparative analysis of temperature-dependent Raman modes in WSe_2_ and MoSe_2_ was presented [28]. Moreover, the study related to the phonon lifetime in WSe_2_ is also limited, among which one prior work reported τ over a range *T* = 4.4–300 K in naturally abundant and isotopically pure WSe_2_ grown by the chemical vapor deposition (CVD) method [29]. In one of our recent works, exciton dynamics and phonon lifetimes in CVD-grown and mechanically exfoliated 1L WSe_2_ nanosheets were analyzed and τ, obtained from temperature-dependent Raman measurements in the 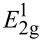 mode, was found to decrease at high temperature due to increased phonon-induced scattering events which eventually also reduces the exciton density in WSe_2_ [30]. In this present work, we studied the phonon lifetime, τ, and phonon concentration in 1L, ML and bulk WSe_2_ using Raman and PL spectroscopy respectively where both the temperature as well as the laser excitation power were varied to better understand the phonon dynamics in 2D WSe_2_ as they are vitally necessary to truly harness its intriguing properties for devices. For example, analysis of the phonon dynamics in 2D WSe_2_ will shed insights on the impact of self-heating effects in WSe_2_ to illustrate its utility in electronic, optoelectronic and thermoelectric device platforms in the future.

In this work, we demonstrate that exposure to heat on the WSe_2_ crystallites as generated via external stimuli such as *T* and *P* causes a red-shift in both the 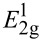 and *A*_1g_ Raman-active modes. Similarly, the FWHM of the Raman peaks is examined which represents the anharmonic terms in the lattice potential energy, and a shift in the peak position and peak broadening effects are noted here as a function of increasing temperature, which is directly related to the phonon damping mechanisms. Finally, we propose a mechanical model to help explain the effect of damping or the rate of decrease of τ as a function of *T* for quantum-confined 1L, as well as ML and bulk WSe_2_.

## Experimental

WSe_2_ nanosheets, including all 1L, ML and bulk samples, were mechanically exfoliated on top of SiO_2_/Si substrates (SiO_2_ thickness = 270 nm) using the scotch tape method [1,30–31]. The mechanical exfoliation process used in this study is similar to the one reported in our earlier work [30]. Prior to the exfoliation procedure, the substrates were cleaned using acetone, isopropyl alcohol and methanol solution in an ultrasonicator, and rinsed with DI water. Nitrogen blow drying and substrate heating on a hotplate at *T* = 110 °C for 5 min allowed any residual moisture from the substrate surface to be removed. The micro-Raman and PL measurements were conducted using a LabRAM HR Evolution NIR (HORIBA Scientific) device equipped with a 532 nm laser for excitation. The micro-Raman and PL spectra were collected by the CCD detector with a diffraction grating of 1800 gr/mm. The spectral resolution for Raman and PL measurements was 0.09 cm^−1^. The Raman band for Si at 520.7 cm^−1^ was used as a reference to calibrate the spectrometer. The laser spot size and spatial resolution were calculated to be ≈2.6 µm and 1.3 µm respectively and the supporting calculations are outlined in Section 1 of the Supporting Information File 1. The WSe_2_ nanosheets were viewed, and the data were collected using a 10× objective (NA = 0.25) of the Raman microscope with working distance 10.6 mm. Due to the instrument limitation, the 10× objective lens could only be used for the temperature-dependent measurement and hence the 10× objective lens was used to collect the data throughout this study. The temperature-dependent Raman and PL measurements were conducted using liquid nitrogen (LN_2_) in a THMS600 Linkam temperature cell at ambient pressure. During the temperature-dependent measurements, the sample was first cooled gradually using LN_2_ from room temperature, i.e., *T* = 298 K to *T* = 79 K after which it was warmed again to *T* = 298 K. Finally heating from room temperature to *T* = 523 K was conducted in a ceramic crucible inside the Linkam cell where a temperature controller enabled the stage to heat the sample.

## Results and Discussion

### Phonon modes in 2D WSe_2_

**Figure 1:**
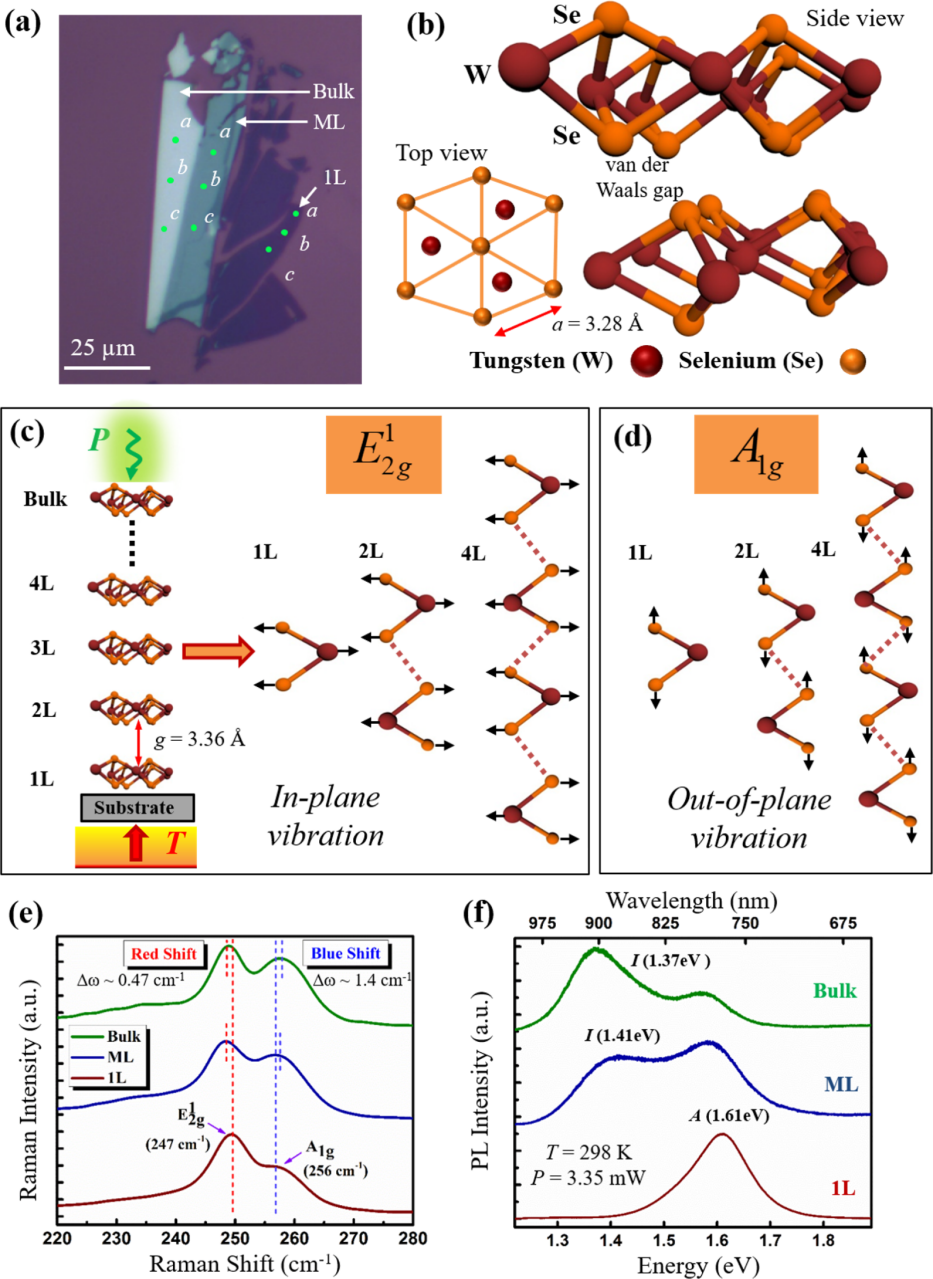
(a) Optical image of monolayer (1L), multilayer (ML), and bulk WSe_2_ nanomembranes. Raman and PL measurements were done on three different sections in the regions marked a, b and c. (b) Schematic representation (top and side views) of the layered structure of (0001) WSe_2_. Schematic representation of frequency evolutions of the (c) 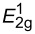 and (d) *A*_1g_ modes for 1L, 2L and 4L of WSe_2_. The layered structure of WSe_2_ on top of a representative substrate is shown in (c)-left, and the application of external stimuli such as laser power *P* and substrate temperature *T*. The interlayer gap *g* is shown as ≈3.36 Å. (e) The variation of the Raman spectra for the 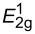 and *A*_1g_ modes for mechanically exfoliated WSe_2_ for 1L, ML, and bulk. The 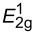 mode exhibits a red-shift Δω ≈ 0.47 cm^−1^ while the *A*_1g_ mode reveals a blue-shift Δω ≈ 1.4 cm^−1^ with increasing layer number. The corresponding error bars calculated from the standard deviation (1σ) of the peak positions in 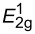 and *A*_1g_ are shown in Supporting Information File 1, Figure S1a. (f) The PL spectra for 1L, ML, and bulk WSe_2_ nanomembranes, where the excitonic *A*-peak represents direct-gap optical transitions, while the *I*-peak is characteristic of indirect band gap optical transitions. The corresponding error bars for the PL *A*- and *I*-peak shifts are shown in Figure S1b.

Mechanically exfoliated high-quality WSe_2_ nanomembranes were observed under an optical microscope through optical contrast differences on the SiO_2_/Si substrates. The optical image of monolayer (1L), ML, and bulk WSe_2_ is shown in Figure 1a. Monolayer TMDCs such as WSe_2_ have a ground state structure with *D*_3_*_h_* symmetry and this phase is called 1H. In bulk structures, the stacking of individual layers results in an alternating rotated sequence which is called the hexagonal symmetric 2H phase that belongs to the inversion-symmetric *D*_6_*_h_* point group. Additionally, WSe_2_ has a lattice constant *a* = 3.28 Å [32] (Figure 1b), and a van der Waals gap *g* = 3.36 Å [33] (Figure 1c, left). The crystal structure of WSe_2_ (0001) consists of a repetition of Se–W–Se trilayers, as depicted in Figure 1b.

The irreducible representation of the phonon modes at the center of the Brillouin zone in WSe_2_ can be described by the following in Equation 1 [7],

[1]
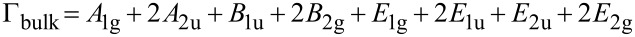


among which *A*_1g_, *E*_1g_, 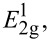 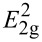 are the Raman active optical modes, 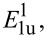 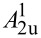 are the acoustic phonon modes, 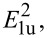 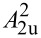 are the infrared active modes, and *E*_2u_, *B*_1u_, 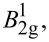 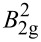 are the four optically inactive modes; however the 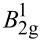 mode is active for bilayer and thicker WSe_2_. In this study we focus on the Raman active modes that are dominant and show a thickness dependence for the 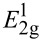 and *A*_1g_ modes.

The respective atomic vibrations related to these two Raman-active modes for 1L and ML WSe_2_ are represented schematically in Figure 1c (right) for the in-plane 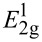 mode and (d) the out-of-plane *A*_1g_ mode, respectively. Additionally, in Figure 1c (left) the layer-wise arrangement of WSe_2_ is shown schematically where the two external stimuli that were used in our experiments, i.e., *P* and *T* are also illustrated.

### Layer-dependent Raman and PL analysis in WSe_2_

As stated previously, the WSe_2_ nanomembranes were mechanically exfoliated from which 1L, ML and bulk regions were identified as shown by the representative case in Figure 1a. Raman and PL spectroscopy was then performed using a Raman laser excitation wavelength of 532 nm on three spatially uniform sections designated 1L, ML, and bulk WSe_2_ with regions a, b and c on the same section marked, as indicated in Figure 1a. Data obtained from repeated measurements in regions a, b, c, served as a means to gauge the standard deviation and the repeatability of the measurements. The error bars thus are calculated from the standard deviation (1σ) of the Raman and PL measurements done on regions a, b and c on the sample for 1L, ML and bulk WSe_2_ which is discussed in detail in Section 2 of Supporting Information File 1. For a fixed laser power (*P* = 3.35 mW) and room temperature (*T* = 298 K), the two Raman-active 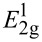 and *A*_1g_ modes were found at ≈247 cm^−1^ and ≈256 cm^−1^, (Figure 1e) for 1L WSe_2_ which is consistent with the previous reports [34–36]. As the thickness of the WSe_2_ increased, the 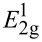 and *A*_1g_ modes shifted in opposite frequency directions; the 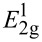 mode experienced a red-shift while the *A*_1g_ mode blue-shifted as thickness increased (Figure 1e). The blue-shift in the *A*_1g_ mode as thickness increased is explained on the basis of the interlayer interaction of the Se atoms in the neighboring planes. From the nearest-neighbor interaction model it is expected, to first-order, that as film thickness increases, a greater restoring force from overlying layers will be present, where the equilibrium average lattice vibrational amplitude is reduced; hence the frequencies of the modes here in the out-of-plane direction will blue-shift as thickness increases [37].

The red-shift of the 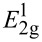 mode as thickness increases is attributed to dielectric screening effects of the long-range Coulomb interaction, where the effective charges resulting from the relative displacement between the W and Se atoms is reduced; this causes the coulombic force to decrease, and hence the energy also decreases [38]. We note from Figure 1e that the blue-shift Δω(*A*_1g_) ≈ 1.4 cm^−1^ is more than three times larger compared to the red-shift 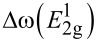 ≈ 0.47 cm^−1^ when the sample thickness increased from 1L to bulk. Another important observation from Figure 1e is the increase in intensity of the *A*_1g_ peak as the number of layers increases, which is likely due to positive reinforcement arising from interference effects for the out-of-plane modes [39]. Prior reports have shown that the difference in peak position, as well as the relative intensities of the 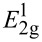 and *A*_1g_ peaks, can serve as a fingerprint for identifying 1L membranes and appears to be a viable platform to decipher material thickness [40–41]. It was found from the data in Figure 1e that the Δω was 7.5 cm^−1^, 8.4 cm^−1^ and 9.1 cm^−1^ for 1L, ML and bulk WSe_2_, respectively. Thus, the Raman peaks exhibit a larger separation as thickness increases.

Monolayer WSe_2_ undergoes a transition from direct to indirect bandgap (*E*_g_) as the number of layers increases [42–43]. We corroborate this here as well with our PL analysis of WSe_2_ for 1L, ML and bulk samples, where Figure 1f depicts a single excitonic *A*-peak at 1.61 eV for 1L which is consistent with the prior reports [35,44]. As the number of layers increases, an indirect peak *I* emerges, which is clearly seen from the PL spectra of the ML and bulk samples. In contrast, the *I* peak is absent in the 1L PL spectra, demonstrating that *E*_g_ undergoes an indirect to direct evolution for the ML to 1L case, respectively. It should also be noted that the *I* peak undergoes a red-shift from the ML to the bulk, and consequently, *E*_g_ was found to be 1.42 and 1.37 eV, respectively, for the two cases.

### Power-dependent Raman and PL analysis in WSe_2_

Besides the above analysis of layer number on the 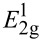 and *A*_1g_ modes, phonon dynamics are also strongly influenced by external stimuli. In this work, we explore two such external stimuli, specifically the Raman laser power *P* and substrate temperature *T* and comment on the light–matter interactions that evolve here in the context of phonon dynamics.

### Power-dependent Raman shifts in WSe_2_

**Figure 2:**
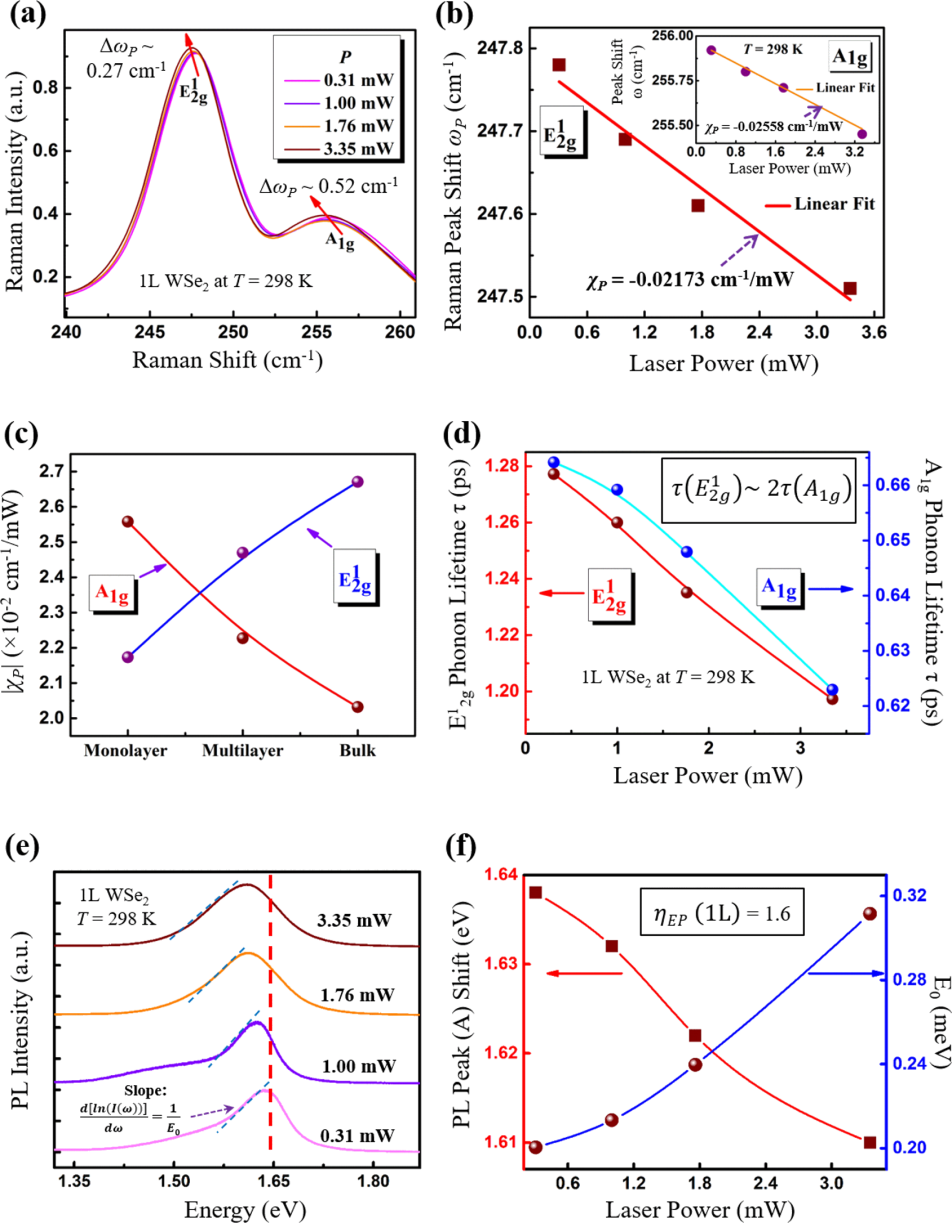
Raman and PL analysis as a function of laser power *P* at *T* = 298 K. (a) Raman spectroscopy of 1L WSe_2_ obtained for *P* in the range of 0.31 mW to 3.35 mW. (b) The Δω_p_ of the 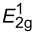 and *A*_1g_ (inset) Raman active modes for 1L WSe_2_ with increasing *P*. The corresponding error bars calculated from the standard deviation (1σ) of the Raman shifts in 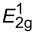 and *A*_1g_ are shown in Supporting Information File 1, Figure S2a. The results were fit linearly from which the slope χ_P_ was computed to be ≈0.02173 cm^−1^/mW and 0.02558 cm^−1^/mW for the 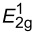 and *A*_1g_ modes, respectively. (c) The variation χ_P_ for the 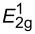 and *A*_1g_ modes for 1L, ML and bulk WSe_2_. (d) Phonon lifetime τ for the 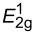 and *A*_1g_ modes as a function of *P* where 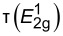 ≈ 2× τ(*A*_1g_) for all *P*. The corresponding error bars calculated for the FWHMs of 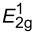 and *A*_1g_ modes are shown in Figure S2b. (e) The normalized PL spectra of the excitonic *A*-peak as a function of *P*. (f) The excitonic *A*-peak position variation as a function of *P* (left-axis) and *E*_0_ (right-axis) representing the phonon concentration that is computed from the low-energy edge of the PL spectra in (e) using Urbach formulism [49] for 1L WSe_2_. The value of η_EP_, calculated from Equation 3, was found to be ≈1.6 for 1L WSe_2_. The corresponding error bars calculated for the PL *A*-peak shift 1/*E*_0_ are shown in Figure S2c.

Starting with the discussion on the laser power, the dependence of the 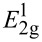 and *A*_1g_ mode in WSe_2_ was analyzed as a function of *P*. Najmaei et al. [45] have analyzed the laser-induced thermal effects in 1L MoS_2_ and with increasing thickness approaching the bulk by means of Raman spectroscopy. Here, for the first time, we report on the variation of the 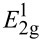 and *A*_1g_ modes for 1L, ML and bulk WSe_2_ crystallites as a function of *P* at *T* = 298 K. In order to minimize the laser-induced effects on the crystallinity and structural changes in the WSe_2_, a laser *P* ≤ 3.35 mW was used. It was also found that the minimum *P* required to get a good signal-to-noise ratio for the Raman and PL data was when *P* ≥ 0.31 mW. Therefore, the only four powers that could be used in this study were 0.31 mW, 1 mW, 1.76 mW and 3.35 mW, i.e., 0.31 mW ≤ *P* ≤ 3.35 mW. This is further explained in detail in Section 3 of Supporting Information File 1. It is also important to note that the *P* was tuned at 0.308 mW first and then it increased gradually to 3.35 mW while the acquisition time was fixed to as low as 0.1 s to further minimize any local heating that could be caused by the laser-induced effects. We termed the change in frequency of the peaks (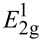 and *A*_1g_) with *P* as Δω_p_. For the 1L case, the red-shift, i.e., Δω_p_ = ω_p_ (*P* = 3.35 mW) − ω_p_ (*P* = 0.31 mW) for both modes was observed, where for the 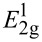, Δω_p_ ≈ 0.27 cm^−1^, and for the *A*_1g_, Δω_p_ ≈ 0.52 cm^−1^ as *P* increased (Figure 2a) for *T* = 298 K. Figure 2b clearly depicts the peak shift Δω_p_ for the 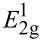 mode as *P* increased from 0.31 mW to 3.35 mW, while the inset shows the corresponding data for the *A*_1g_ mode. The Δω_p_ is characterized by Equation 2 below [27],

[2]
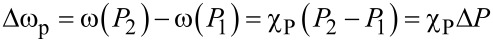


where χ_P_ is denoted as the power-dependent Raman coefficient and is obtained from the slope of the first-order fit to the Δω_p_ versus *P* plot (Figure 2b). The fitted coefficients χ_P_ for the 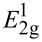 and *A*_1g_ modes were computed to be −0.02173 cm^−1^/mW and −0.02558 cm^−1^/mW, respectively. The variation of χ_P_ for 1L, ML and bulk WSe_2_ is plotted in Figure 2c, which clearly shows χ_P_ for the 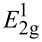 mode increases while it decreases for the *A*_1g_ mode.

### Phonon lifetime from power-dependent Raman analysis in WSe_2_

As mentioned earlier, phonon dynamics in 2D TMDCs also involves the determination of the phonon lifetime τ. The phonon lifetime τ is determined from the FWHM, i.e., phonon linewidth (Γ) of the 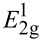 and *A*_1g_ peaks in the Raman spectra. The measured Raman linewidths are a convolution of effects of both the actual Lorentzian vibrational distribution of the phonons and the instrument-induced broadening which is typically assumed to have a Gaussian response and is provided by the Voigt profile [46]. The actual Γ was therefore determined from the Voigt profile fitted to the experimental data, illustrated in Figure S4 for 1L WSe_2_ at *T* = 298 K in Section 4 of Supporting Information File 1.

The instrumental broadening Δυ, calculated to be 0.613 cm^−1^, is lower compared to the measured phonon linewidths Γ in the Raman active 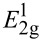 and *A*_1g_ modes from our analysis and hence, the influence of instrumental broadening on the calculation of phonon lifetimes from the phonon linewidth of the Raman active modes can be considered as negligible. The approach used to calculate the instrumental broadening is discussed in detail in Section 4 of Supporting Information File 1*.* As *P* increased, Γ was seen to increase based on the broadening of the Raman peaks for dispersive or dissipative media. As *P* increased, the incoming optical radiation thermalizes the lattice vibrations in both modes. Given the asymmetric nature of the lattice potential energy plot, a larger mean spacing between atoms arises at the higher effective temperature which increases the amplitude of the phonon vibrations as well as their potential for scattering events [20,47].

By using the energy uncertainty relationship with the phonon linewidth [46] we calculated τ using,

[4]
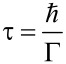


where τ is in picoseconds, and ℏ is the modified Planck’s constant (5.3 ps·cm^−1^). The τ for the 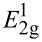 and *A*_1g_ modes is depicted in Figure 2d, where τ decreased as *P* increased due to more damping in the phonon vibrations caused by the increased likelihood of scattering events. We define a parameter, the power-dependent phonon lifetime ratio η_τP_ as given by Equation 5,

[5]
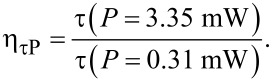


For the 1L case η_τP_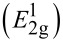 and η_τP_(*A*_1g_) was determined to be 0.954 and 0.945, respectively. Another artifact from this data in Figure 2d is the important observation that 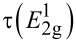 ≈ 2 × τ(*A*_1g_) for all of the values of *P* measured. This appears to corroborate that the coupling in the *A*_1g_ mode between layers is rather weak given the larger separation; however, for the in-plane vibrations the phonon modes are well coupled and hence the phonon lifetime in the in-plane direction is larger when compared to the out-of-plane direction.

### Phonon concentration from power-dependent PL analysis in WSe_2_

The other aspect of phonon dynamics is the phonon concentration and the change in phonon concentration in WSe_2_ due to change in excitation energy, which has been analyzed in this study. The PL measurement in 1L WSe_2_ as *P* increased at *T* = 298 K (Figure 2e) depicts the variation of the excitonic *A*-peak, representing direct-gap transitions, where *E*_g_ (1L) = 1.61 eV. The shift of the *A*-peak towards lower energies as *P* increased is further illustrated in Figure 2f (left axis), while a broadening of the lower energy side of the PL peak in Figure 2e was also observed as *P* increased. Urbach [48] discussed this dependence of spectrum in the context of various types of excitation sources used which formed the basis for the so-called Urbach formulism. Ramos and Luzzi [49] used this Urbach formulism to explain the behavior displayed in the radiation emission band of semiconductors at high-excitation levels, and the slope of the low-energy edge of the spectrum was characterized with an empirical parameter *E*_0_ on a semi-logarithmic plot. This parameter *E*_0_ has a dimension of energy and varies with excitation energy [49]. Ko et al. [50] also showed that the value of *E*_0_ increases with *P* and *T* in 1L MoS_2_ as a result of excess phonons generated when the system is perturbed beyond equilibrium. Similarly, in this study, from this low-energy edge due to excitons [51] and using Urbach formulism, the slope of the low-energy edge of the excitonic *A*-peak (Figure 2e) is calculated by the following Equation 6 [49],

[6]
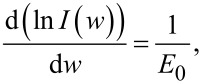


where *I*(ω) is the intensity, and *E*_0_ is in units of meV and is the so-called characteristic energy parameter [50], which is correlated to the phonon concentration. The variation of *E*_0_ (right-axis) as a function of *P* is shown in Figure 2f, which indicates that the phonon concentration increases with increasing *P*. This is likely due to the excess phonons generated above equilibrium due to the higher effective temperature as *P* increases [50]. Once again, we define a parameter, the power-dependent energy parameter ratio η_EP_, given by Equation 3,

[3]
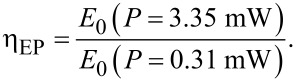


The value of η_EP_ was calculated to be 1.6 which implies that *E*_0_ at *P* = 3.35 mW is 1.6 × *E*_0_ at *P* = 0.31 mW for 1L WSe_2_. Again, this increment in *E*_0_ is understandable due to the increase in phonon concentration as a result of increasing laser excitation energy.

### Temperature-dependent Raman and PL analysis in WSe_2_

The temperature-dependent Raman analysis was carried out using a temperature cell where LN_2_ was used for cooling down to ≈79 K and a heater within the temperature cell allowed the temperature to reach 523 K. During the cooling process, it was found that the signal to noise ratio was very poor when both the *T* and *P* became very low, particularly in case of PL measurements. The best signal to noise ratio in both the Raman and PL measurements was found for *P* = 3.35 mW even when *T* was as low as ≈79 K and therefore the power was fixed at 3.35 mW throughout the temperature-dependent measurements in this study. It is also important to mention here that during the heating process, the intensity of the Raman signal started decreasing and above *T* > 523 K, no signal was detected, which may be attributed to material degradation arising in WSe_2_ through the possibility of an increased point defect density at higher temperatures, and/or through oxidative effects. From our preliminary checks on the samples, the Raman spectrum does not recover once the temperature is ramped down to 298 K. This indicates irreversible changes that may have transpired in the WSe_2_ as a result of the thermal cycle. In contrast, in the cooling down cycle from 298 to 79 K, and the subsequent warming back up cycle to 298 K, the Raman data was analyzed for any hysteresis effects. This data is shown in Section 5 of the Supporting Information File 1, Figure S5a and S5b for the 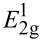 and *A*_1g_ modes in 1L WSe_2_, respectively in Section 5 of Supporting Information File 1. The largest difference in the Raman shift arising from hysteresis was found to be ≈0.21 cm^−1^ (*T* = 173 K) and 0.15 cm^−1^ (*T* = 248 K) for 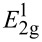 and *A*_1g_ modes, respectively, suggesting that hysteresis is negligible for measurements at 298 K and below.

### Temperature-dependent Raman shifts in WSe_2_

**Figure 3:**
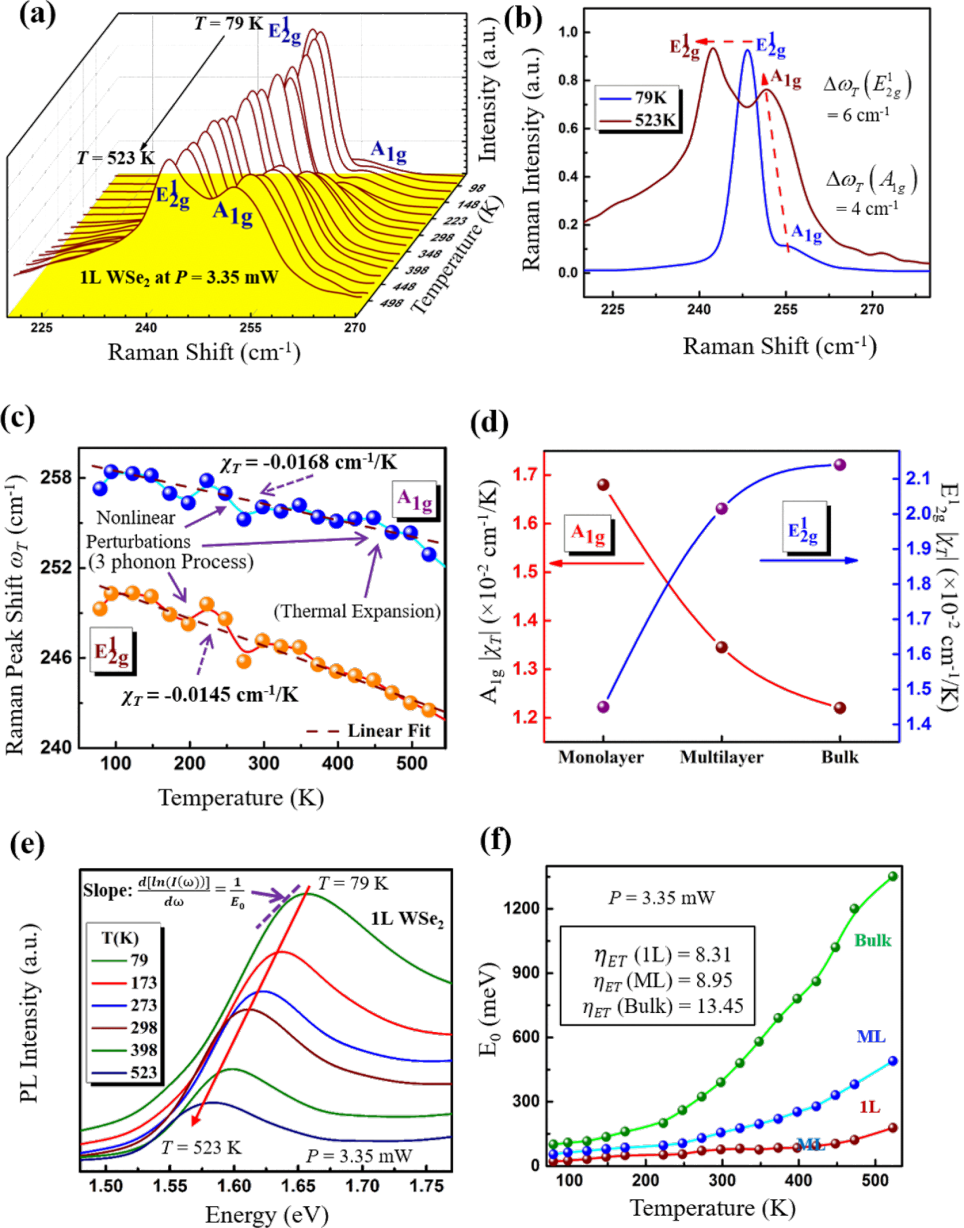
Raman and PL analysis as a function of temperature *T* for *P* = 3.35 mW. (a) Raman spectroscopy of 1L WSe_2_ obtained for temperature in the range from 79 to 523 K. The corresponding error bars calculated from the standard deviation (1σ) of the Raman shifts and FWHM in 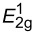 and *A*_1g_ modes are shown in Supporting Information File 1, Figure S2d and Figure S2e, respectively. (b) The net change in Δω_T_ and intensity of the 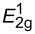 and *A*_1g_ modes from *T* = 79 K to *T* = 523 K. The net red-shift Δω_T_ was found to be ≈6 cm^−1^ and 4 cm^−1^ for the 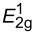 and *A*_1g_ modes, respectively. (c) The frequency shift, Δω_T_ of the 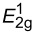 and *A*_1g_ Raman active modes for 1L WSe_2_ with increasing *T*. The results were fit linearly using Equation 7 from which the slope χ_T_ was computed to be about −0.0145 cm^−1^/K and −0.0168 cm^−1^/K for the 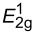 and *A*_1g_ modes, respectively. The nonlinear perturbation of Raman shift for the 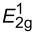 mode mainly originates from the anharmonic effect of three-phonon processes, whereas for the *A*_1g_ mode it is determined mainly by the three-phonon process in the low-*T* regime and in the high-*T* regime it is dominated by the thermal expansion effect. (d) The variation of χ_T_ for the 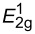 and *A*_1g_ modes with different layers of WSe_2_. (e) The normalized PL spectra of the excitonic *A*-peak as a function of T in 1L WSe_2_. (f) The excitonic *A*-peak position was used to calculate *E*_0_ and represents the phonon concentration that is computed from the low-energy edge of the PL spectra in (e) using Urbach’s formulism [49] for 1L WSe_2_. The phonon concentration *E*_0_ is shown for 1L, ML and bulk WSe_2_. The values of η_ET_, calculated using Equation 8, were found to be 8.31, 8.95 and 13.45 for 1L, ML and bulk WSe_2_, respectively. The corresponding error bars calculated for the PL *A*-peak shift 1/*E*_0_ are shown in Figure S2f.

Figure 3a shows the temperature-dependent Raman spectra for 1L WSe_2_ for the 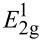 and *A*_1g_ modes. The observed changes in phonon frequencies with *T* in 1L WSe_2_ is attributed to the asymmetry in the interatomic potential versus displacement function [47], which leads to a larger average equilibrium lattice spacing. As the lattice expands or contracts with *T*, the equilibrium position of atoms and consequently the interatomic equilibrium separation changes, which induces a shift in the phonon energies. This behavior of the Raman peak frequencies with *T* is seen in many materials as *T* changes, from which a thermal expansion coefficient can be deduced [52–53]. Similar to the *P* dependence, here we termed the change in frequency of the peaks with *T* as Δω_T_. As seen from Figure 3b, the red-shift of Δω_T_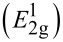 was ≈6 cm^−1^ as *T* increased from 79 to 523 K, while its intensity remains constant. Similarly, the red-shift of Δω_T_(*A*_1g_) was ≈4 cm^−1^; however the intensity of the *A*_1g_ mode increased significantly with increasing *T*. This is likely due to the out-of-plane vibrations of the Se atoms that may be less constrained by the substrate for the *A*_1g_ mode. In contrast, the in-plane vibrations of the 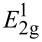 mode are likely to be more constrained by the substrate at the Se-substrate interface even though temperature increased [54], and so the intensity of the 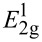 mode did not increase significantly. In one of our prior reports [30], similar trends were observed in the intensities of 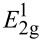 and *A*_1g_ mode in CVD-grown 1L WSe_2_. However, the Δω_T_ in 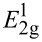 and *A*_1g_ modes were found to be less (Δω_T_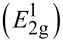 ≈ 5.10 cm^−1^, Δω_T_(*A*_1g_) ≈ 3.54 cm^−1^) in the case of CVD grown 1L WSe_2_ [30], which could be attributed to the different film preparation methods used in both the studies [24].

The Se–substrate interface constraint may be assumed to be negligible in bulk WSe_2_. However, we noticed that the intensity of 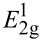 mode in bulk WSe_2_ (Supporting Information File 1, Figure S6) also remained less sensitive with temperature. This is likely due to the fact that the Se–substrate interface may not be the only constraining factor and there may be some other underlying mechanisms operative here, such as the the thermal properties of the substrate itself, or the sample preparation approach used, which could influence the vibrational properties and consquently the intensity of the in-plane 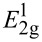 mode in WSe_2_. Su et al. reported on the influence of the substrate, film morphology, and film preparation method on the Raman active modes in 1L, 2L and bulk MoS_2_ and showed that the 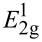 mode in MoS_2_ is modified more strongly if an epitaxial sapphire substrate is used compared to SiO_2_/Si substrates [24]. As the SiO_2_/Si susbstrate and the preparation method (mechanical exfoliation) remained the same for 1L and bulk WSe_2_ used in our study, this may be the reason why the intensity of the 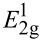 Raman mode remained unchanged even in bulk WSe_2_. Moreover, in this temperature-dependent study, thickness was confirmed on representative 1L and bulk WSe_2_, as shown by the data in Figure S7 of Supporting Information File 1. The change in peak position of the 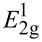 and *A*_1g_ modes with increasing *T* is plotted in Figure 3c. The results were fit to the Grüneisen model which was originally formulated for diamond [55], and is also applicable here, as given by Equation 7 below,

[7]
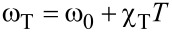


where ω_0_ is the frequency of vibration of the 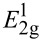 and *A*_1g_ modes at *T* = 0 K and χ_T_ is the first-order Raman temperature coefficient calculated for both the 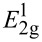 and *A*_1g_ modes (just as in the computation of χ_P_, the slope of the linear fit represents the value of χ_T_). The values for χ_P_ and χ_T_ for ML and bulk samples of WSe_2_ are listed in Table 1. The χ_T_ for the 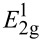 and *A*_1g_ modes was computed to be −0.0145 cm^−1^/K and −0.0168 cm^−1^/K, respectively, which appear to be comparable to those reported for WSe_2_ [28] and other exfoliated TMDCs, such as MoS_2_ [22–23,56], MoSe_2_ [28]_,_ and WS_2_ [25]. The thickness dependence of χ_T_ for both the 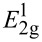 and *A*_1g_ modes is plotted in Figure 3d, and it was found that χ_T_ for the *A*_1g_ mode decreased, while on the other hand, it increased for the 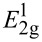 mode as the layer number increased. This dependence was similar to the variation noted for χ_P_ with layer thickness, as discussed earlier.

**Table 1:** Magnitudes of *T*- and *P*-dependent coefficients for the 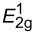 and *A*_1g_ Raman-active modes and comparison to prior results reported for other TMDCs. Here χ_T_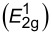 represents the first-order temperature-coefficient for 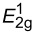; χ_T_(*A*_1g_) represents the first-order temperature-coefficient for *A*_1g_; χ_P_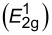 represents the first-order power-coefficient for 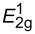; and χ_P_(*A*_1g_) represents the first-order power-coefficient for *A*_1g_. The χ_T_ and χ_P_ increased for the 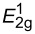 mode as the layer number increased, while a decrease for the *A*_1g_ mode was noted.

Material	|χ_T_| 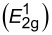 (cm^−1^/K)	|χ_T_| (*A*_1g_) (cm^−1^/K)	|χ_P_| 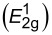 (cm^−1^/mW)	|χ_P_| (*A*_1g_) (cm^−1^/mW)	Ref.

WSe_2_ (1L)	0.0145	0.0168	0.02173	0.02558	this work
WSe_2_ (ML)	0.02015	0.01345	0.0247	0.02271	this work
WSe_2_ (bulk)	0.0214	0.0122	0.02671	0.02032	this work
WSe_2_ (1L)	0.0048	0.0032	–	–	[26]
MoSe_2_ (1L)	–	0.0054	–	–	[28]
WS_2_ (1L)	0.006	0.006	–	–	[25]
MoS_2_ (1L)	0.0124	0.0143	–	–	[22]
MoS_2_ (ML)	0.0132	0.0123	–	–	[23]
MoS_2_ (bulk)	0.0147	0.0123	–	–	[56]

Incidentally, the temperature-dependent Raman active 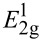 and *A*_1g_ modes actually exhibited some nonlinearity in certain regions that are described as nonlinear perturbations in Figure 3c. It was observed that the nonlinear perturbations of the Raman shift in the *A*_1g_ mode were present over the entire temperature regime, whereas for the 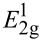 mode, a distinctively linear region is presented in the high-*T* regime above ≈350 K. To analyze the physical origin of these nonlinear perturbation, a physical model [26] is invoked as expressed by Equation 9,

[9]
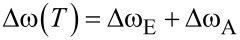


where Δω_E_ is the Raman shift change induced by lattice thermal expansion, which leads to a red-shift as discussed earlier. Additionally, the Δω_A_ is the Raman shift attributed to anharmonic effects of the three- and four-phonon process. A light scattering process can be assumed to be comprised of: the absorption of a photon, the emission of a photon, and the creation of an optical phonon which then decays anharmonically into two phonons, three phonons, etc. The production of two and three phonons is known as a three-phonon and four-phonon process, respectively. The nonlinear perturbations in the 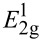 mode mainly originate from the anharmonic effect of three-phonon processes (Figure 3c), whereas for the *A*_1g_ mode, there is a contribution from the thermal expansion effect as well as in the high-*T* regime given the out-of-plane *A*_1g_ vibrations that are less constrained by the substrate. However, the three-phonon anharmonic effect still presides in the low-*T* range for the *A*_1g_ mode [26]. As a result of this the nonlinear perturbations were found to be persistent in the *A*_1g_ mode even at high temperature. Additionally, the laser absorbance efficiency in WSe_2_ will change with temperature, which could also affect the phonon shifts in the 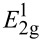 and *A*_1g_ modes, as found in Figure 3c.

### Phonon concentration from temperature-dependent PL analysis in WSe_2_

Photoluminescence spectroscopy was carried out similarly as the temperature was varied at fixed *P* = 3.35 mW for 1L, ML and bulk WSe_2_ samples. The effect of temperature on the broadening and red-shift of the direct-band excitonic *A*-peak in 1L WSe_2_ is shown in Figure 3e. At high temperature, the phonon concentration increases, and the phonon scattering results in the broadening of exciton radiation [56]. This increased phonon scattering also causes the phonon lifetime to decay at high temperature, which is discussed in the next section. Additionally, the intensity of the PL *A*-peak also decreases at high temperature due to the temperature-induced enhancement of the exciton–phonon coupling and nonradiative recombination [57]. The change in phonon concentration was measured from *E*_0_ according to Equation 6, and the variation of *E*_0_ with *T* for 1L, ML and bulk samples is shown in Figure 3f. The energy parameter, *E*_0_, increases exponentially with *T* and its value is much higher for ML and bulk samples when compared with 1L samples across the entire temperature range shown in Figure 3f. Similar to η_EP_, we define a parameter, the temperature-dependent energy parameter ratio η_EP_, given by Equation 8,

[8]
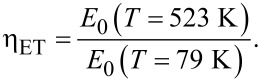


The values of η_EP_ (1L), η_ET_ (ML), η_ET_ (bulk) were found to be 8.31, 8.95 and 13.45, respectively, which confirms the fact that the phonon concentration is higher in the bulk compared to the 1L case as *T* increased from 79 to 523 K. It is not surprising that in increasing temperature, more phonon modes are excited, which in turn increases the phonon concentration and hence the value of *E*_0_. The value of η_ET_ (1L) > 5× η_EP_ (1L), which is possibly due to the more direct coupling of energy into the system by thermal means, in contrast to the local variation caused by an increase in the laser power where the energy coupling and the optothermal transduction may not be as effective. The following fit in Equation 10 was applied to the *E*_0_(*T*) data as a function of *T*,

[10]
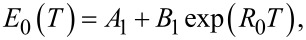


where *A*_1_, *B*_1_ are constants having dimension of energy, and *R*_0_ is the exponential increment rate of *E*_0_. From the fitting, the value of *R*_0_ (1L) for WSe_2_ was found to be ≈0.0017/K, which is almost 3× < *R*_0_ (ML) and *R*_0_ (bulk), as shown by the comparative values in Table 2. This indicates the rate of increment in phonon concentration is much smaller in 1L WSe_2_ when compared with ML and bulk samples as temperature increases. This is again consistent with intuitive considerations where a larger number of phonon modes are available and excited in the 3D bulk state since phonon density increases as *T^3^*, while in a quantum confined system such as the 1L case, the phonon density is not as strong a function of *T*. This also has a direct influence on the thermal conductivity of quantum-confined 1D structures where the thermal conductivity should be high and not vary substantially with *T*, as has already been deduced through experimental verifications of thermal conductivity in MoS_2_ [23,27] and other layered materials [58].

**Table 2:** Determination of *R*_0_ and *D*_0_ which correspond to the increment rate of phonon concentration and the decay rate in phonon lifetime, respectively, with increasing *T.* The *R*_0_ and *D*_0_ values decreased by 3× and 2×, respectively for 1L when compared to bulk WSe_2_.

WSe_2_ layers	*R*_0_ (/K)	*D*_0_ (/K) 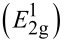

1L	0.0017	0.0031
ML	0.00487	0.0047
bulk	0.00557	0.0063

### Phonon lifetime from temperature-dependent Raman analysis in WSe_2_

**Figure 4:**
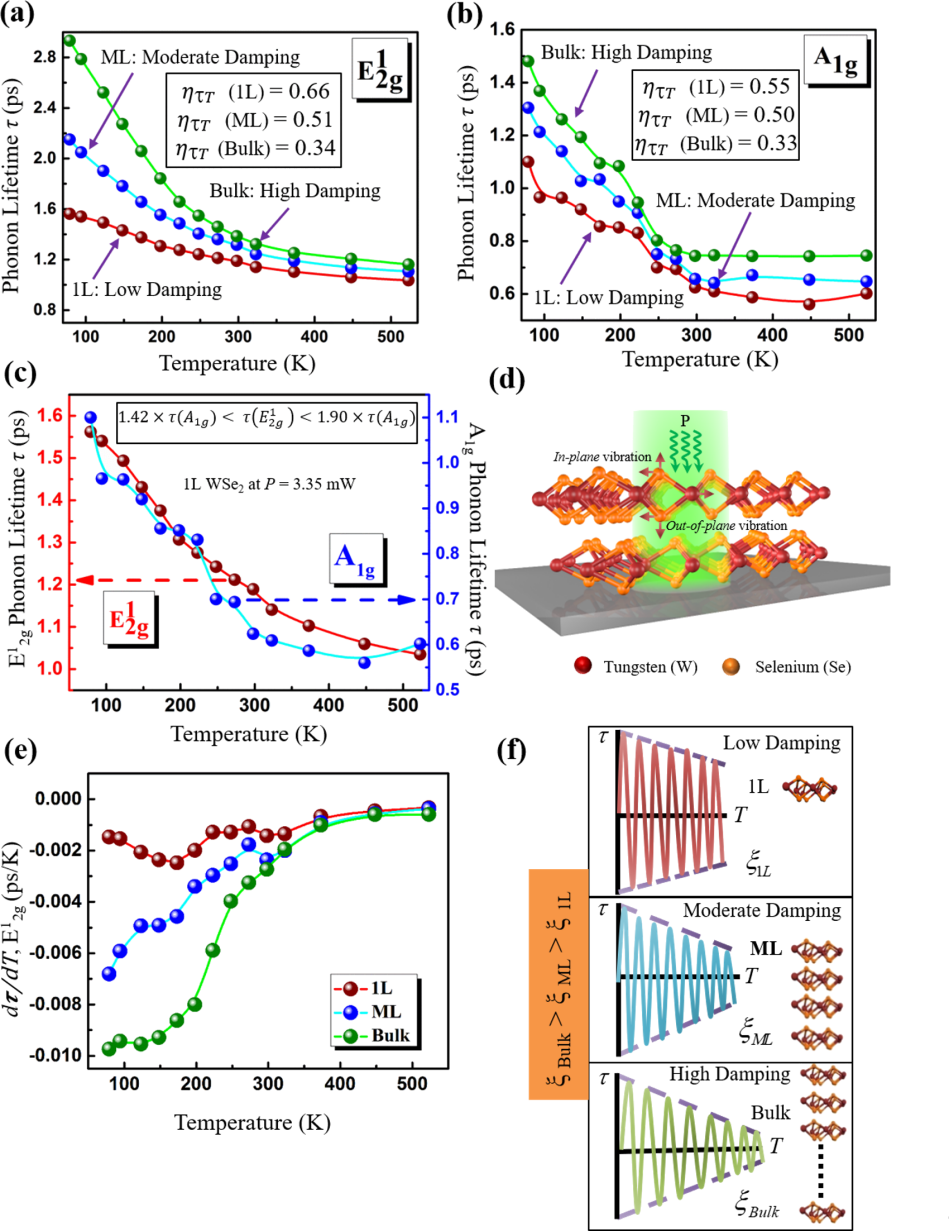
The phonon lifetime τ variation with *T*. Temperature-dependent phonon lifetime variation in 1L, ML and bulk WSe_2_ nanomembranes for the (a) 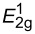 and (b) *A*_1g_ modes. The value of η_τT_, calculated using Equation 11 for 1L, ML and bulk WSe_2_ samples yielded 0.66, 0.51 and 0.34 for 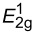 mode and 0.55, 0.50 and 0.33 for *A*_1g_ mode, respectively. (c) Phonon lifetime τ for the 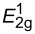 and *A*_1g_ modes as a function of *T*. It was found that 1.42 × τ(*A*_1g_) < 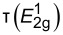 < 1.90 × τ(*A*_1g_) by taking the maximum and minimum ratio of 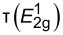 over τ(*A*_1g_). (d) The proposed 3D schematic representation of our analysis which shows the effect of external excitation, in this case the incoming laser power, in the vibrational modes in 2D layered WSe_2_. (e) The rate of change in τ with respect to *T* for the 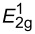 mode in 1L, ML and bulk WSe_2_. (f) A damped harmonic oscillator model is proposed to qualitatively represent the low, moderate and high damping scenarios in the WSe_2_ samples, where ξ_1L_, ξ_ML_ and ξ_bulk_ are the damping coefficients associated with 1L, ML and bulk WSe_2_, respectively. From the phonon concentration and decay rate data as a function of WSe_2_ thickness, it was concluded that ξ_1L_ < ξ_ML_ < ξ_bulk_ as shown by the three scenarios: ξ_1L_(left), ξ_ML_(middle), ξ_bulk_(right).

The observed broadening of the Raman peak in WSe_2_ with increasing *T* arises from damping of the excited optical phonon, and the line width Γ is inversely proportional to the phonon lifetime τ which is calculated using Equation 4. Figure 4a and 4b show the variation of τ for 1L, ML and bulk samples with increasing *T* for the 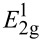 and *A*_1g_ modes, respectively. It is clear from this data that τ decreases for all sample thicknesses as *T* increases, similar to the dependence of τ on *P*, as shown by the data in Figure 2d. This decrease occurs as the rate of phonon–phonon scattering events increases with *T* due to the associated increase in the phonon thermal occupancy and hence, their interaction [47]. The phonon lifetime for 1L WSe_2_ was found to be ≈1.19 ps for the 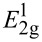 mode (Figure 4a) at *T* = 298 K which is consistent with the value of τ(≈1.2 ps) in 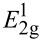 mode in ME 1L WSe_2_ reported in our prior work [30]. It has been also found that τ is higher in sapphire substrate (≈1.4 ps) due to the decreased interfacial defect density and built-in strain on sapphire that reduces the electron–phonon interaction in the material [30]. The value of τ obtained in our study is also comparable to what has been reported for τ in 1L CVD grown WSe_2_ (≈0.76 ps) [30], bilayer CVD grown WSe_2_ (≈2.4 ps) [29], MoS_2_ nanoparticle (≈1.4 ps) [59], and 1L graphene (≈1.2 ps) [60]. We compared our results with the phonon lifetimes of conventional bulk 3D materials such as GaN [38,61], AlN [61], ZnO [61] and the values are provided in Table 3. Similar to τ_P_, here we also define a define a parameter, the temperature-dependent phonon lifetime ratio η_τT_ as given by Equation 11 below,

[11]
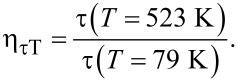


The values of η_τT_ for 1L WSe_2_ were found to be 0.66 and 0.55 for the 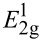 and *A*_1g_ modes, respectively. The value of η_τT_ is less for both Raman modes when compared with η_τP_, which is likely due to more scattering occurring over the wider temperature range, when compared with the laser power variation.

**Table 3:** Comparison of τ in 2D TMDCs and conventional bulk 3D materials. From our work, the τ for the 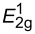 mode was found to be almost 2× higher compared to the *A*_1g_ mode for 1L, ML and bulk WSe_2_.

Material	Mode	Phonon lifetime (τ) (ps)	Ref.

WSe_2_ (1L)	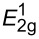 , *A*_1g_	1.19, 0.62	this work
WSe_2_ (ML)	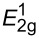 , *A*_1g_	1.32, 0.65	this work
WSe_2_ (bulk)	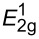 , *A*_1g_	1.38, 0.74	this work
WSe_2_ (1L)	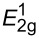 (SiO_2_/Si, sapphire)	1.2, 1.4	[30]
WSe_2_ (2L)	*E*_2g_, 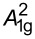	2.4, 1.3	[29]
MoS_2_ (nanoparticle)	*E*_1u_	1.4	[59]
graphene	G-Band	1.2	[60]
graphite	G-Band	2.4	[60]
GaN (bulk)	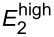	2.56	[44]
GaN (bulk)	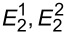	10.1, 1.4	[61]
AlN (bulk)	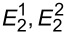	4.4, 0.83	[61]
ZnO (bulk)	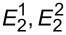	5.9, 0.9	[61]

The values of η_τT_ for both the 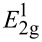 and *A*_1g_ modes in ML and bulk samples were found to be very similar, as is evident from the data in Figure 4a and 4b. The variation of τ with *T* in 1L WSe_2_ for both 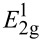 and *A*_1g_ modes is depicted in Figure 4c where it was found that 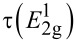 > τ(*A*_1g_) for all temperatures, which is quite similar to what we found in Figure 2d where we showed 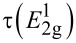 ≈ 2× τ(*A*_1g_) for all *P*. Specifically, the upper and lower bounds for τ were determined to be 1.42 × τ(*A*_1g_) < 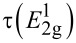 < 1.90 × τ(*A*_1g_) for all *T*. The decay in τ caused by the thermal excitation energy is shown schematically in Figure 4d; here the effect of external stimuli, such as the energy of incoming photons from the laser beam, on the vibrational modes of the atoms in WSe_2_ is illustrated. The external energy causes damping in the in-plane 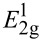 mode and the out-of-plane *A*_1g_ mode within the WSe_2_ crystal, which affects the phonon frequencies and subsequently their lifetime and concentration.

### Phonon lifetime and decay mechanisms in WSe_2_

Another perspective that elucidates the dependence of τ with *T* is the rate at which τ decays with *T*. As seen from Figure 4a and 4b, the rate of decay in τ in 1L, ML and bulk WSe_2_ varies widely in low *T*, but remains almost constant at high *T*. This is because the decay in τ is governed by various scattering mechanisms which are caused by the interaction of phonons with defects, boundaries, other phonons, etc. It is important to analyze the scattering as a whole through the analysis of the individual scattering events themselves, as deduced from Matthiessen’s rule [38,46,62]. As discussed earlier, the phonon linewidth broadening arises from the scattering of phonons with defects, doping, electrons, etc., and consequently, the resulting τ is an average of the contributions from each of those scattering sources. Hence, the phonon concentration, defect density, doping concentrations, etc. determine the effective strength of a scattering source and disparities between them cause the differences in the measured lifetimes in 1L, ML and bulk WSe_2_ which we observed in Figure 4a and 4b. Interestingly, the defect-induced scattering events will not dominate the overall scattering mechanisms as the defect concentration will not change significantly due to the stable crystal arrangement in WSe_2_ over the course of the measurements. However, this is not the case for other scattering events such as phonon–phonon scattering. According to the Bose–Einstein distribution, the population of phonons will change with *T*, which will in turn influence the number of phonon–phonon scattering events [46]. Thus, the phonon concentration increases with *T* (Figure 3f) and consequently τ will be dominated by phonon–phonon scattering at high *T*. Figures 4a and 4b also show that τ decays similarly in 1L, ML and bulk WSe_2_ at higher *T* which may be explained through a dominant source common to all samples. To confirm this, we examined the rate of change in τ with respect to *T*, i.e., 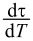 for the Raman active 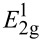 mode in 1L, ML and bulk WSe_2_ which is shown in Figure 4e. It was found that at high *T* there was a convergence of 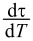, indicating that the phonon–phonon scattering was dominant in all the specimens which governed the decay mechanism at high *T*. In contrast, at low *T*, τ (Figure 4a and 4b) and consequently 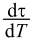 (Figure 4e) vary widely in 1L, ML, and bulk WSe_2_, as nanostructure differences become the more dominant scattering source at low *T*. These dissimilarities in the decay in 1L, ML and bulk WSe_2_ at low *T* are characterized by fitting the results of Figure 4a, i.e., τ(*T*) for the 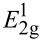 mode, to the exponential decay function as noted in Equation 12,

[12]
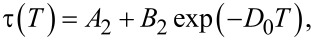


where *A*_2_, *B*_2_ are constants having units of time and *D*_0_ is the decay rate in τ with units of K. The value of *D*_0_ (1L) was ≈0.0031/K while the *D*_0_ (bulk) was ≈0.0063/K which is almost 2× higher; the data are once again summarized in Table 2.

We conceptualize these results using a mechanical harmonic oscillator model to qualitatively compare the decay in τ with *T* due to damping mechanisms governed by phonon–phonon scattering as discussed above in WSe_2_ as a function of sample thickness. Figure 4f depicts this visually, where three different damping scenarios are illustrated. Based on our data in Figure 4a and Figure 3f, the damping is low, moderate and high in 1L (top schematic in Figure 4f), ML (middle schematic in Figure 4f) and bulk samples (bottom schematic in Figure 4f), respectively, since the rate of decay of τ increases from 1L to bulk samples. This further confirmed the result we observed earlier from the PL analysis of WSe_2_ with varying *T* (Figure 3f) where it was found that the rate of increment *R*_0_ in phonon concentration with increasing *T* is lowest for 1L and highest for bulk samples, suggesting that the mean free path for scattering between phonons decreases, which increases the probability of collisions when the phonon concentration increases, eventually decreasing phonon lifetime. We compared our phonon lifetime τ and temperature *T* to a proposed model of the standard harmonic oscillator exhibiting damping with, 
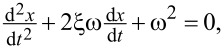
 where *x* represents displacement of the oscillator, *t* is the time and ξ is the damping coefficient which we figuratively relate to the three scenarios for 1L, ML and bulk as ξ_1L_, ξ_ML_ and ξ_bulk_, respectively. Thus, from the damping in the tabulated τ values, the three damping ratios, ξ_1L_, ξ_ML_ and ξ_bulk_ are shown in Figure 4f. It is clear from our analysis and from the values of *D*_0_ (Table 2) that ξ_1L_ < ξ_ML_ < ξ_bulk_, i.e., the damping is least for the 1L case and it is the highest for bulk WSe_2_. Understanding the phonon dynamics and factors such as phonon lifetime, decay rate and nonradiative decay pathways, such as defects, will prove to be vital for truly harnessing the unique properties of WSe_2_ and designing high-performance electronic, optoelectronic, thermal, and thermoelectric devices from WSe_2_ in the future.

## Conclusion

In conclusion, we report, the dependence of the Raman and PL of 1L, ML and bulk WSe_2_ by external stimuli such as laser power and temperature, from which we analyze the phonon lifetime and change in phonon concentration in WSe_2_. Our results show that both temperature and laser power affect the phonon dynamics in WSe_2_. It was found that the phonon concentration increases, followed by a decrease in the phonon lifetime, for increasing thickness of WSe_2_ with increasing energy of the external stimuli (temperature and laser power). We also analyzed the first-order temperature- and power-dependent 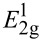 and *A*_1g_ coefficients and found that the coefficients increase for the 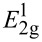 mode and decrease for the *A*_1g_ mode as the number of layers increases. A comparative analysis of the phonon frequencies and their relative changes from 1L to bulk WSe_2_ samples was conducted, which showed a frequency and intensity shift for 1L, ML, and bulk samples. The mechanical model we proposed provides a framework in which to explain the damping mechanisms in WSe_2_ with varying thickness and the increase in energy from external stimuli. The insights from this work reveal the importance of light–matter interactions in 2D WSe_2_ to alter the phonon spectrum toward the quantum-confined limit of monolayers. These findings can be broadly applied to other layered materials to help guide the design of high-performance electronic, optoelectronic, thermal, and thermoelectric devices based on WSe_2_ in the future.

## Supporting Information

The supporting information includes: Section 1: Calculation of laser spot size, Section 2: Calculation of error bars for Raman & PL measurements, Section 3: Optimization of laser powers used in Raman and PL measurements, Section 4: Calculation of instrumental broadening and its effect on phonon lifetime analysis, Section 5: Calculation of hysteresis during cooling down and warming up processes, Section 6: Temperature-dependent Raman spectra in bulk WSe_2_, and Section 7: AFM, Raman and PL characterization of 1L and bulk WSe_2_.

File 1 Additional material.
