# Supplementary material for: Light–matter interactions in two-dimensional layered WSe2 for gauging evolution of phonon dynamics
Source: Beilstein J Nanotechnol. 2020 May 12;11:782–97. doi: 10.3762/bjnano.11.63 (PMC7237805; doi:10.3762/bjnano.11.63)
Supplement: File 1 — Additional material. [file Beilstein_J_Nanotechnol-11-782-s001.pdf]

BEILSTEIN JOURNAL OF NANOTECHNOLOGY

Supporting Information

for

Light–matter interactions in two-dimensional layered WSe_2 for gauging evolution of phonon dynamics

Avra S. Bandyopadhyay, Chandan Biswas and Anupama B. Kaul

Beilstein J. Nanotechnol. **2020,** *11,* 782–797. doi:10.3762/bjnano.11.63

Additional material

License and Terms: This is a supporting information file under the terms of the Creative Commons Attribution License (http://creativecommons.org/licenses/by/4.0). Please note that the reuse, redistribution and reproduction in particular requires that the authors and source are credited.
The license is subject to the *Beilstein Journal of Nanotechnology* terms and conditions: (https://www.beilstein-journals.org/bjnano)

1. Calculation of laser spot size

The laser spot size is primarily defined by the laser wavelength and microscope objective using Rayleigh's criterion. The laser spot diameter *R* is calculated using the following:

$$R = \frac{1.22\lambda}{NA} \quad (1)$$

where λ is the wavelength of the Raman laser and NA is the numerical aperture of the microscope objective. For our study, the wavelength of the laser was 532 nm and for a 10× microscope objective, the NA was 0.25. Hence, using Equation 1 the laser spot diameter *R* was calculated to be ≈2.6 µm. A slightly modified equation yields the theoretical diffraction limited spatial resolution which is achievable using an optical microscope, and is given by the following formula:

$$Spatial\ resolution = \frac{0.61\lambda}{NA} \quad (2)$$

After putting the respective values of wavelength and NA in Equation 2, the spatial resolution was found to be ≈1.3 µm. However, in Raman spectroscopy, scattering of the laser photons and interaction with the interfaces in the sample can reduce the resolution. Thus, typical spatial resolution in the Raman process is more than the theoretical calculated value.

2. Calculation of error bars for Raman & PL measurements

The Raman and PL measurements were done over a wide range of temperatures on WSe_2 comprised of multiple layer thicknesses. Making multiple measurements on the same spot of the sample to extract reproducibility data and generate error bars over the course of the temperature and sample thickness experiments was deemed practically challenging and perhaps not yielding the true impact of the external temperature on the Raman spectra. The laser–sample interactions could unduly heat the local area of the molecular membranes during repeated and successive measurements which could potentially induce defects and affect the structural morphology of the WSe_2 nanosheets, convoluting the Raman spectra. Therefore, in order to extract error-bars in our

data, we choose three spatially uniform regions marked as *a*, *b*, and *c* on the sample in each of the 1L, ML, and bulk WSe_2 sections, as shown in Figure 1a of the manuscript. For Raman and PL spectroscopy measurements, the mean value for measurements conducted in *a*, *b* and *c* were calculated from which the standard deviation (1σ) were determined in order to extract the error bars.

The Raman and PL characteristics in 1L, ML and bulk WSe_2 at a fixed temperature (T = 298 K) and laser power (P = 3.35 mW) are shown in Figure 1e and 1f in the manuscript, respectively. The error bars, i.e., standard deviation (1σ) calculated for the Raman shift of Raman active E_{2g}^1 and A_{1g} modes from Figure 1e are shown in Figure S1a. Similarly, Figure S1b shows the error bars calculated for *A*- and *I*-peak in PL characteristics from Figure 1f.

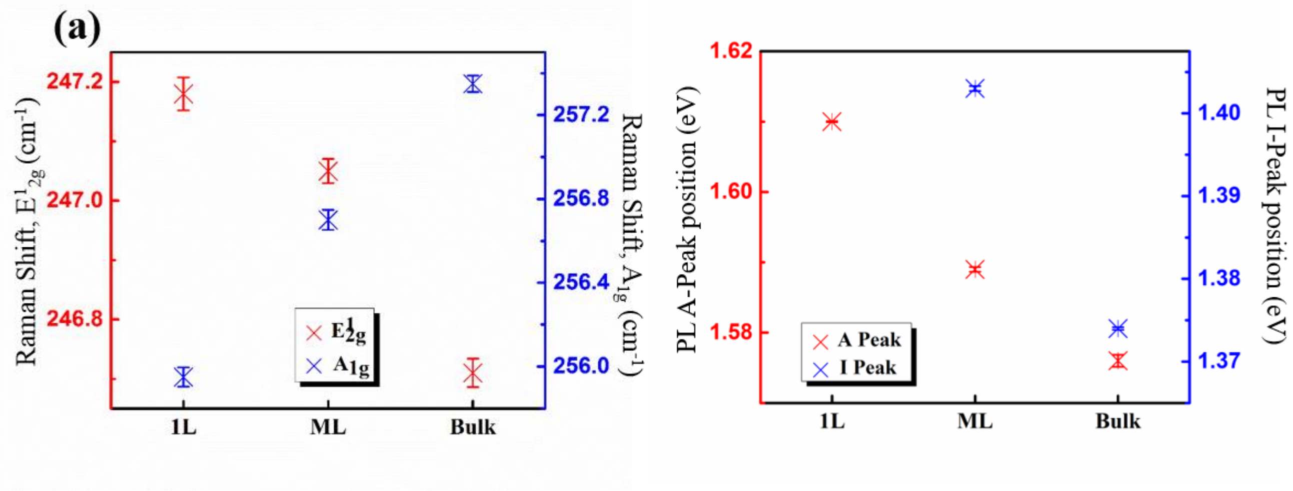

Figure S1: The error bars extracted from 1σ of the (a) Raman shifts in the E_{2g}^1 and A_{1g} modes in the Raman spectra (Figure 1e), and (b) *A*- and *I*-peak shifts in the PL spectra (Figure 1f) of 1L, ML and bulk WSe_2.

The Raman and PL characteristics in 1L WSe_2 at a fixed temperature (T = 298 K) with varying laser power (P = 0.308 mW to P = 3.35 mW) are shown in Figure 2 of the manuscript. The laser power coefficients and lifetime of the Raman active E_{2g}^1 and A_{1g} modes are calculated using the

Raman shifts and FWHM of E_{2g}^{1} and A_{1g} modes, respectively. The error bars for the Raman shifts and FWHM of E_{2g}^{1} and A_{1g} modes calculated from Figure 2a of the manuscript are shown in Figure S2a and Figure S2b respectively. The shift in the *A*-peak and the energy parameter E_0 are calculated from the position and the lower energy slopes ($1/E_0$) of the *A*-peak in the PL spectra in 1L WSe_2, respectively. The error bars for the PL *A*-peak shifts and $1/E_0$ are calculated from Figure 2e of the manuscript and are plotted in Figure S2c.

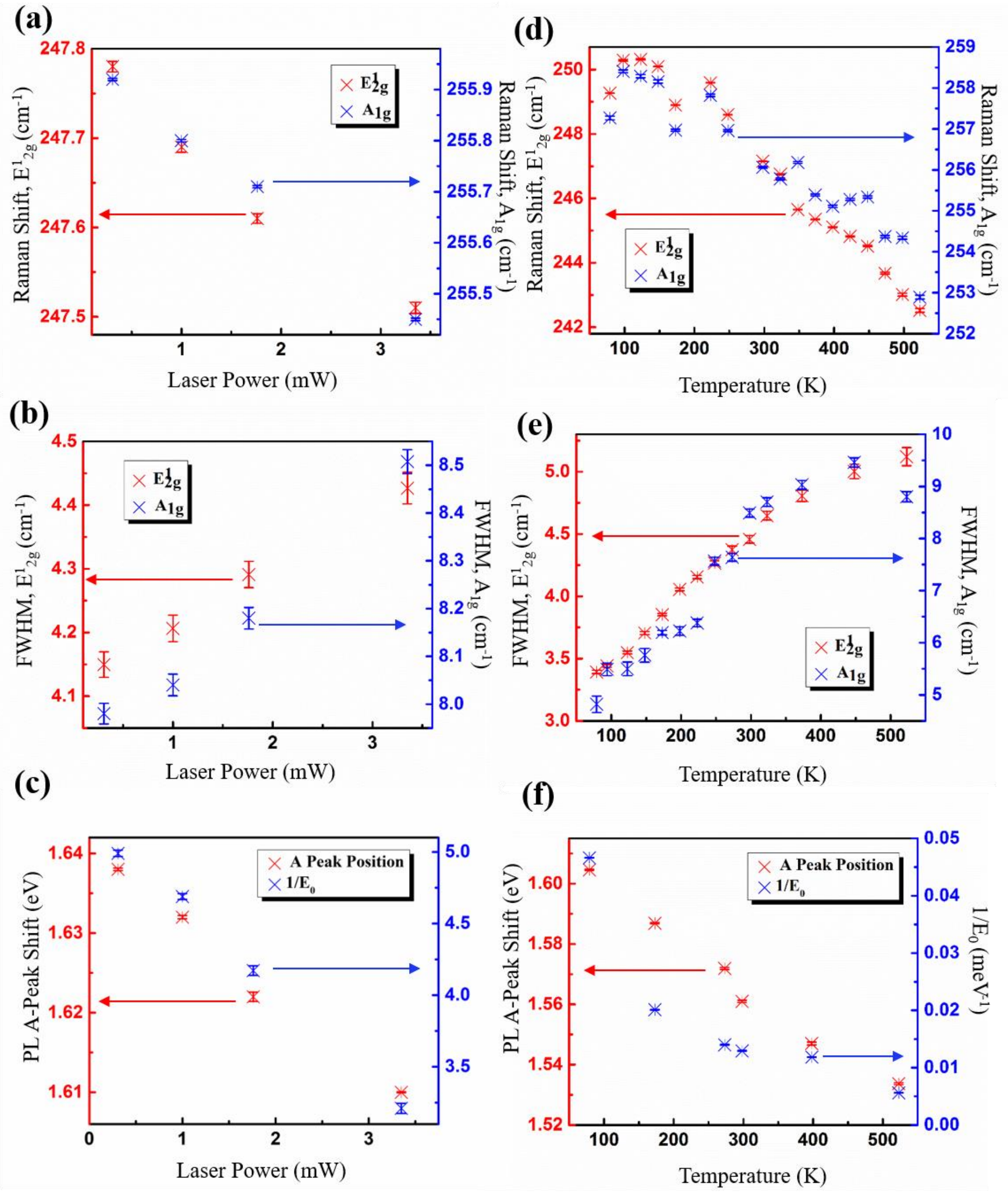

Figure S2: The error bars extracted from 1σ of the (a) Raman shifts and (b) FWHM in E^1_{2g} and A_{1g} modes in the Raman spectra (Figure 2a) and for (c) PL A peak shift and $1/E_0$ in PL spectra (Figure 2e) in 1L WSe_2 at a fixed T = 298 K and varying P (P = 0.308 mW to P = 3.35 mW). The error bars extracted from 1σ of the (d) Raman shifts and (e) FWHM in E^1_{2g} and A_{1g} modes in Raman spectra (Figure 3a) and (f) PL A-peak shift and $1/E_0$ in PL spectra (Figure 3e) in 1L WSe_2 at a fixed P (3.35 mW) and varying T (T = 79 K to T = 523 K).

The Raman and PL characteristics in 1L WSe_2 at a fixed laser power (P = 3.35 mW) and varying temperature (T = 79 K to T = 523 K) are shown in Figure 3 in the manuscript. Similar to the analysis of the laser power variation study as discussed above, the error bars for the Raman shifts and FWHM of E^1_{2g} and A_{1g} modes calculated from Figure 3a of the manuscript are shown in Figure S2d and S2e respectively. The error bars for the PL A-peak shifts and $1/E_0$ are calculated from Figure 3e of the manuscript and are plotted in Figure S2f.

Figure 4a, 4b and 4c in the article shows the lifetimes of the Raman active E^1_{2g} and A_{1g} modes for a wide range of T which are calculated from the FWHM of the respective Raman modes and the error bars for the corresponding measurements in 1L WSe_2 is already shown in Figure S2e as discussed.

3. Optimization of laser power used in Raman and PL measurements

In our study, the laser power P was tuned using a ND filter in LabRAM HR Evolution (Horiba Scientific) spectrometer. The calculated laser powers corresponding to the tunable ND filters are given in Table S1 from which it can be inferred that the minimum achievable P was 0.00325 mW when the laser was tuned with 0.01% of the ND filter.

Table S1: The calculated P for different values of ND filters in Horiba LabRam HR Evolution spectrometer used in our study.

ND Filter (%)	Calculated Power (mW)
0.01	0.00325
0.1	0.0243
1	0.308
3.2	1
5	1.76
10	3.35
25	9
50	17
100	34

The Raman and PL signals were measured at the minimum P = 0.00325 mW in monolayer (1L), multilayer (ML) and bulk samples. In case of 1L samples a decent signal to noise ratio (SNR) was observed; however, the SNR was found to be very poor when bulk WSe_2 was measured under such low laser power. For example, Figure S3 shows below the PL spectra of 1L and bulk WSe_2 nanosheets when the ND filter was tuned at 0.01% which reveals that the SNR is very low in case of bulk WSe_2 while it is decent in 1L nanosheets.

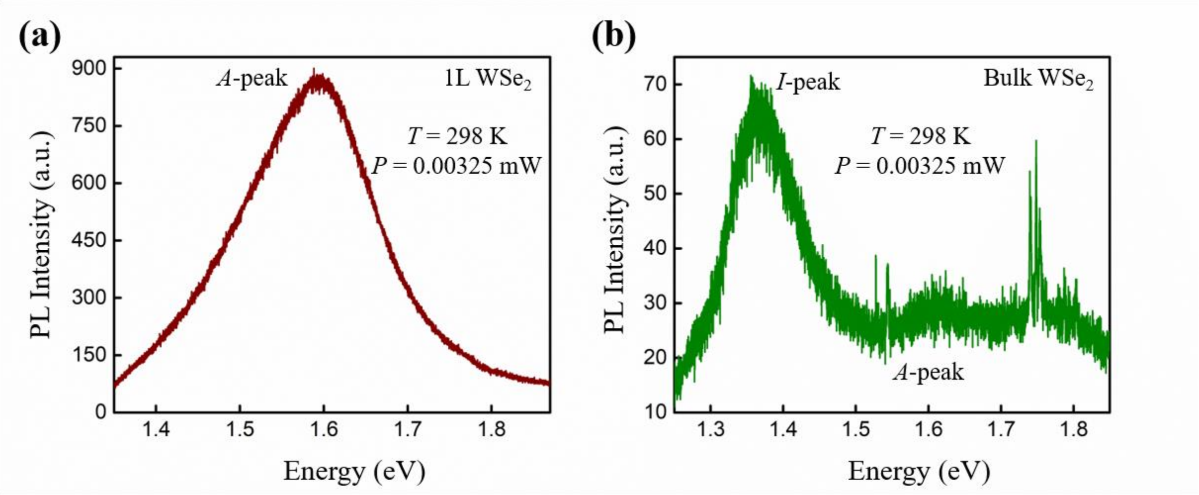

Figure S3: The PL spectra in (a) 1L and (b) bulk WSe_2 when P is tuned at the minimum possible value of 0.00325 mW. The PL spectra in 1L WSe_2 shows a good SNR. However, bulk WSe_2 shows very poor SNR and the *A*-peak is not distinguishable from noise in the PL spectra.

It was found that SNR only improves in bulk WSe_2 from P = 0.308 mW (ND filter = 1%) and that is why it was the minimum P chosen throughout the study as we found it was beneficial to use a common minimum laser power to compare and analyze different parameters in the 1L, ML and bulk nanosheets.

4. Calculation of instrumental broadening and its effect on phonon lifetime analysis

In our study, phonon lifetimes τ of the Raman active E_{2g}^{1} and A_{1g} modes in 1L, ML and bulk WSe_2 nanosheets are calculated from the full-width-half-maximum (FWHM), i.e., phonon linewidth Γ of E_{2g}^{1} and A_{1g} modes by using Equation 3 described in the manuscript. The measured Raman linewidths are a convolution of effects of both the actual Lorentzian vibrational distribution of the phonons and the instrument-induced broadening which is typically assumed to have a Gaussian response. The convolution of the phonon line shape and the spectrometer function is known as the Voigt profile [1]. The true phonon linewidth Γ was therefore determined from the Voigt profile fitted to the experimental data illustrated for 1L WSe_2 at T = 298 K in Figure S4. The instrumental broadening was calculated to be 0.613 cm^{-1} which is far lower compared to Γ in E_{2g}^{1} and A_{1g} modes and it is discussed in more detail below.

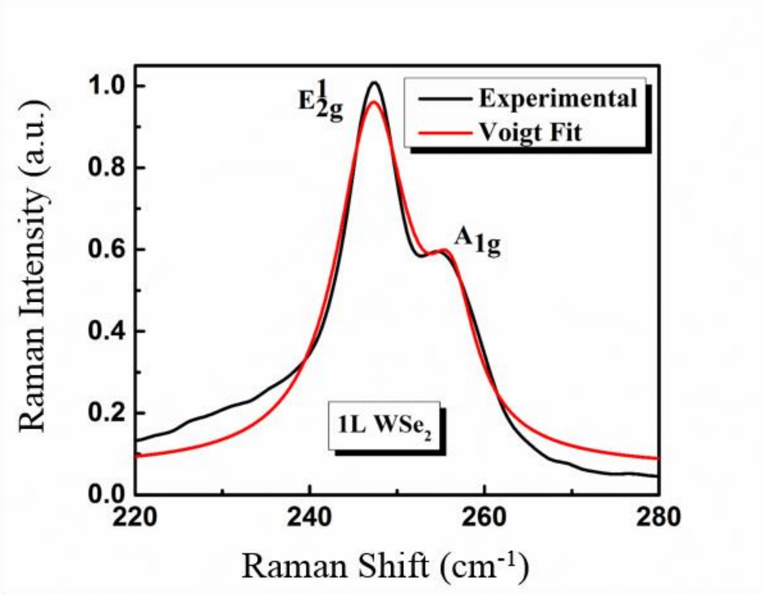

Figure S4: Raman Spectra of 1L WSe_2 at T = 298 K. The experimental data is fitted with the Voigt profile to calculate the phonon linewidth Γ.

The calculation of instrumental broadening is described below:

It is understood that the condition for constructive interference and obtaining a primary maximum for a diffraction grating Raman spectrometer used in our study is given by,

$$m\lambda = d(sin(\theta_i) + sin(\theta_r)) \quad (3)$$

where λ is the wavelength (532 nm) of the incident Raman laser, *m* is the order, *d* is the slit spacing, θ_i and θ_r are the angle of incidence and reflection respectively. Here, we assume that $\theta_i = \theta_r = \theta$, order $m = 1$, and $\sin\theta \approx \theta$, for very small value of θ. Hence, Equation 1 can be rewritten as, $\Delta\lambda \approx d\Delta\theta$, where Δλ is the instrument broadening, i.e., change in λ for a small change in θ or Δθ.

A slit width *b* will introduce a $\Delta\theta = \frac{b}{f}$, where *f* is the focal length of the spectrometer; for our spectrometer *f* is 800 mm. This results in,

$$\Delta\lambda = \frac{db}{f} \quad (4)$$

There is a lower limit that can be achieved by narrowing the slit width, $\frac{\Delta\lambda}{\lambda} > \frac{1}{Nm}$, where *N* is the number of lines on the grating that are used in creating the spectral line. However, there is an issue in narrowing the slit width, as it also lowers the signal-to-noise ratio. In order to satisfy these conflicting requirements *b* was chosen as 25 μm for our measurements.

By putting $d = \frac{1}{1800}$ mm (as the laser grating is 1800 gr/mm) in Equation 4 we calculated the value of the instrumental broadening $\Delta\lambda \approx 0.017$ nm.

The wavenumber υ is related to through $\upsilon = \frac{1}{\lambda}$, and by taking derivative with respect to λ, we have the following instrumental broadening in terms of υ as,

$$\Delta\upsilon = \frac{\Delta\lambda}{\lambda^2} \quad (5)$$

By using the noted values in Equation 5, we determine that $\Delta\upsilon \approx 0.613\ cm^{-1}$. The experimentally obtained values of the Γ in our study ranges from $3.393 \pm 0.014\ cm^{-1}$ to $5.120 \pm 0.073\ cm^{-1}$ for the E_{2g}^{1} mode and $4.819 \pm 0.158\ cm^{-1}$ to $8.802 \pm 0.099\ cm^{-1}$ for the A_{1g} modes in 1L WSe_2 for the T-dependent Raman measurements from which τ calculated using Equation 3 as described in the main text. These results show that $\Delta\upsilon << \Gamma$, and hence the influence of instrumental broadening on the calculation of phonon lifetimes from the FWHMs of the Raman active modes was negligible in our study.

In the case of the PL measurements, we found that the FWHM is 60 nm for 1L WSe_2 while Δλ was found to be 0.017 nm from Equation 4, as described above. Therefore, similar to the Raman measurements, the effect of instrumental broadening on the FWHM of the PL peak was also minimal in our study.

5. Calculation of hysteresis during cooling down and warming up processes

The Raman shift of the Raman active E_{2g}^{1} and A_{1g} modes in 1L WSe_2 during cooling to T = 79 K and then warming to T = 298 K are shown in Figure S5a and S5b respectively.

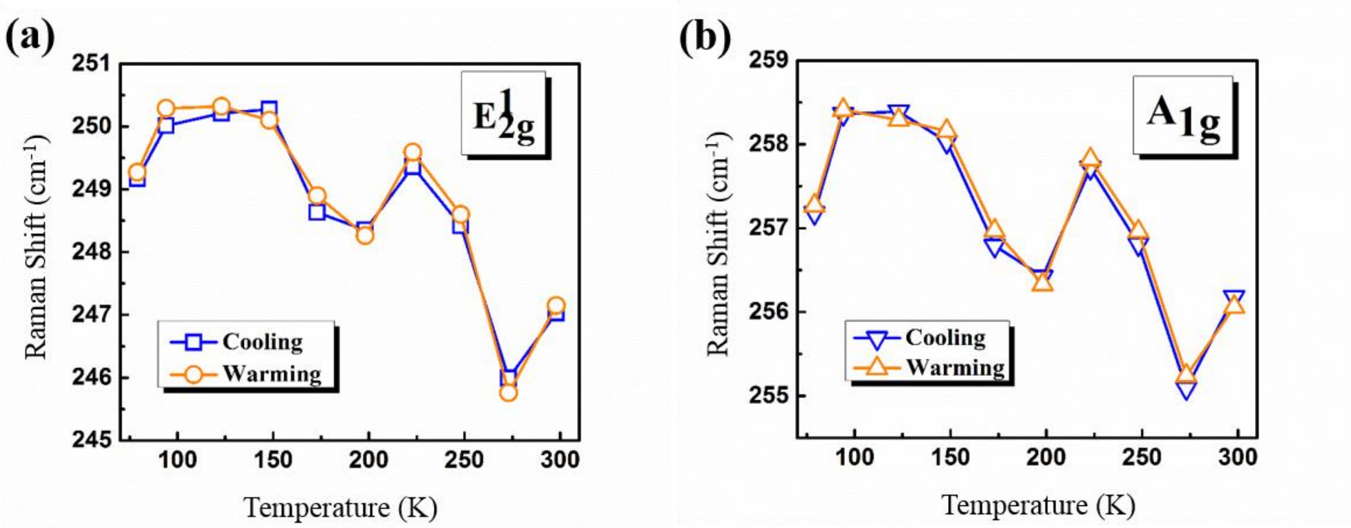

Figure S5: The Raman shift in the (a) E_{2g}^{1} and (b) A_{1g} modes during cooling (T = 298 K to T = 79 K) and warming up cycle (T = 79 K to T = 298 K).

6. Temperature-dependent Raman spectra in bulk WSe_2

The T-dependent Raman spectra for E_{2g}^{1} and A_{1g} modes in bulk WSe_2 samples is shown in Figure S6a. The intensity of the E_{2g}^{1} mode in bulk WSe_2 also remained less sensitive with T similar to that in 1L WSe_2 which is shown in Figure 3a. The intensity ratio of the Raman active A_{1g} mode to that of E_{2g}^{1} mode is plotted for a wide temperature range for 1L, ML and bulk WSe_2 in Figure S6b which shows that the intensity ratio is higher in ML and bulk WSe_2 compared to that in 1L WSe_2.

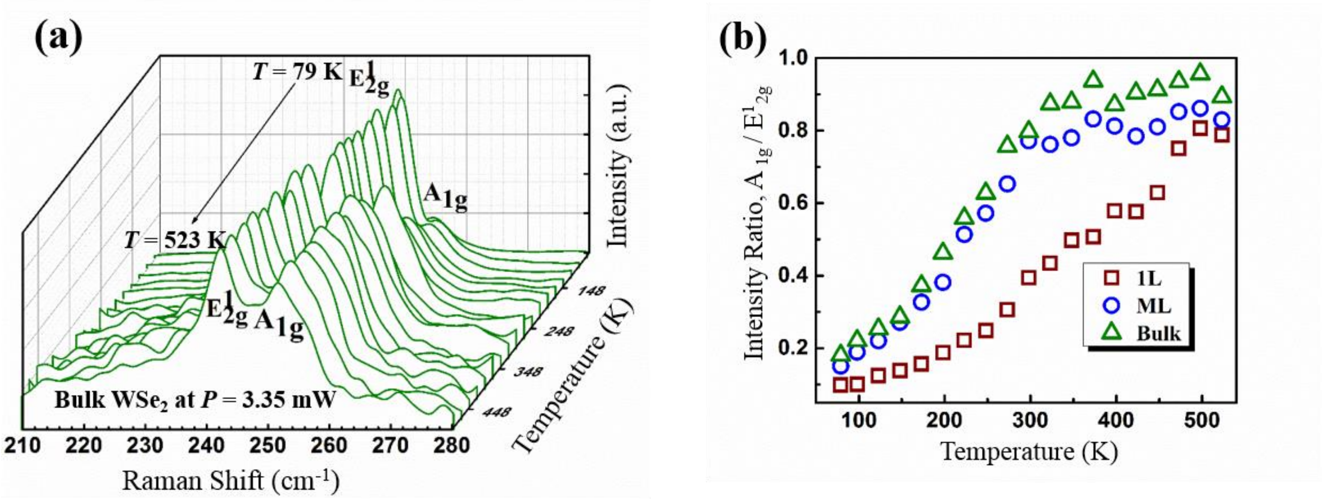

Figure S6: (a) Temperature dependent Raman spectra in bulk WSe_2 and (b) A_{1g}/E_{2g}^{1} Intensity ratio variation A_{1g}/E_{2g}^{1} in 1L, ML and bulk WSe_2.

7. AFM, Raman and PL characterization of 1L and bulk WSe_2

The optical images of an ultrathin WSe_2 mechanically exfoliated on SiO_2/Si, is depicted in Figure S7a in which the 1L WSe_2 is highlighted by the color contrast. The AFM characterization was done using Bruker Multimode 8 AFM with Nanoscope V controller at *University of North Texas, Denton*. Figure S7b depicts the AFM image of the ultrathin WSe_2 which shows a thickness from the scan direction of A to B of ~ 0.77 nm which is consistent with other reported values of thickness in single layer 2D TMDCs [2-4]. Similarly, Figure S7c and d show the optical and AFM images of a bulk WSe_2 from which the step height in the direction of C to D was calculated to be ≈6 nm. The Raman and PL characterization of these samples are shown in Figure S7e and f.

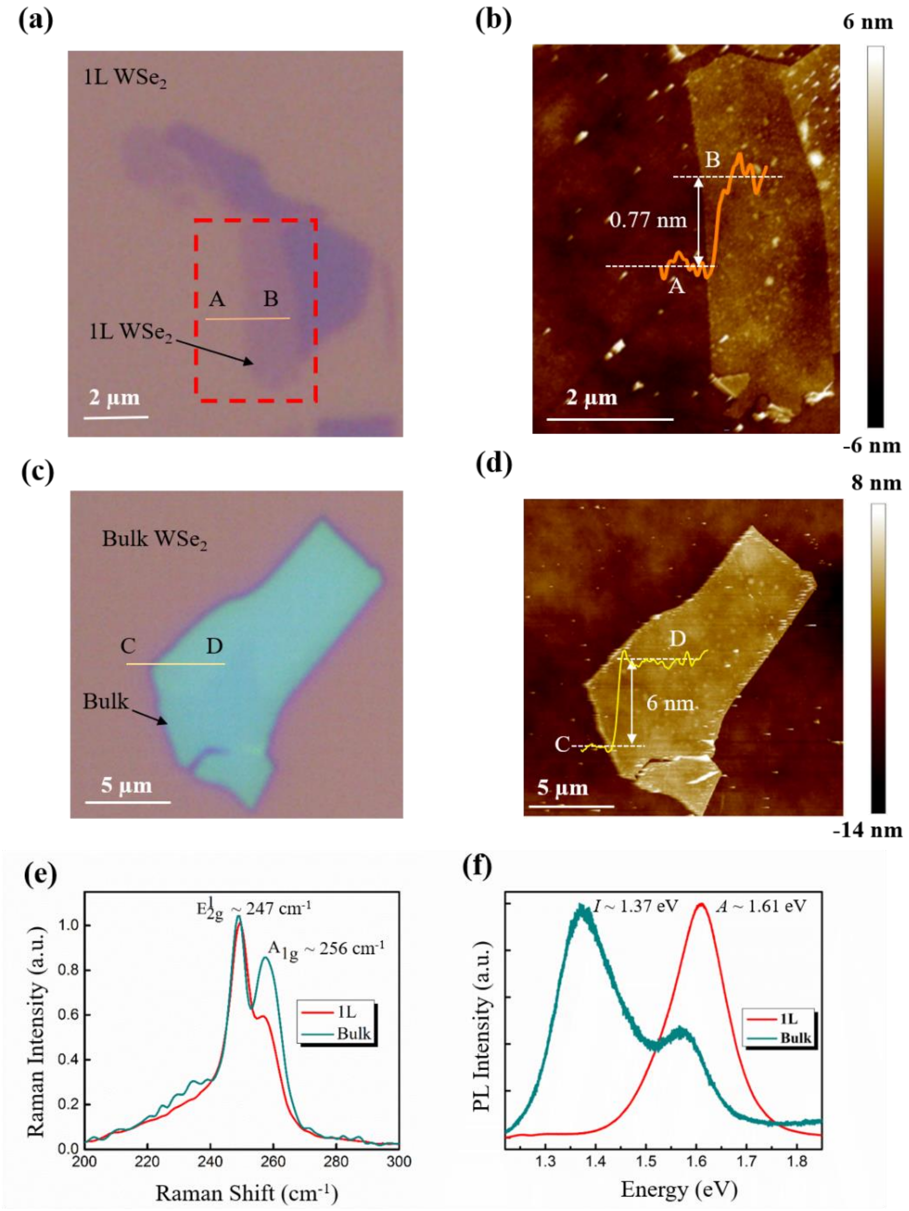

Figure S7: (a) The optical image of an ultrathin WSe_2 nanomembrane. The red box indicates the region where the AFM measurement is done. (b) The AFM image shows a step height in the A to B scan direction of 0.77 nm which is consistent with single layer thickness attributed to 2D TMDCs [2-4]. (c) The optical image of a bulk WSe_2. (d) The AFM image of the bulk WSe_2 shows a step height in the C to D scan direction ≈6 nm. (e) The Raman characteristics of 1L and bulk WSe_2 show the characteristics E_{2g}^{1} and A_{1g} peaks at ≈247 cm^{-1} and 256 cm^{-1} respectively. (f) The PL characteristics show the *A*-peak at 1.61 eV in 1L WSe_2 and *I*-peak at 1.37 eV in bulk WSe_2 corresponding to direct bandgap and indirect bandgap emission respectively. These results are consistent with the results showed for 1L and bulk WSe_2 in Figure 1e and 1f of the manuscript.

The two Raman active E_{2g}^1 and A_{1g} modes which characterize WSe_2, were found at ≈247 cm^{-1} and 256 cm^{-1} respectively. The intensity of the E_{2g}^1 mode remains nearly constant in both 1L and bulk WSe_2, but the intensity of A_{1g} mode found to be very small in 1L WSe_2 compared to bulk, a phenomenon which is consistent with the results shown in Figure 1e of the main text. Moreover, the PL spectrum shows (Figure S6f) the *A*-peak at 1.563 eV in 1L and *I*-peak at 1.368 eV bulk WSe_2 corresponding to direct bandgap and indirect bandgap emission respectively which are again consistent with the PL characteristics shown in Figure 1f in the manuscript.

8. References

1. Beechem, T.; Graham, S. *J. Appl. Phys.* **2008,** *103*, 093507. doi:10.1063/1.2912819

2. Yan, R.; Simpson, J. R.; Bertolazzi, S.; Brivio, J.; Watson, M.; Wu, X.; Kis, A.; Luo, T.; Walker, A. R. H.; Xing, H. G. *ACS Nano* **2014,** *8*, 986–993. doi:10.1021/nn405826k

3. Late, D. J.; Shirodkar, S. N.; Waghmare, U. V.; Dravid, V. P.; Rao, C. N. R. *ChemPhysChem* **2014,** *15*, 1592–1598. doi:10.1002/cphc.201400020

4. Najmaei, S.; Liu, Z.; Ajayan, P. M.; Lou, J. *Appl. Phys. Lett.* **2012,** *100*, 013106. doi:10.1063/1.3673907
